# The Cancer Chimera: Impact of Vimentin and Cytokeratin Co-Expression in Hybrid Epithelial/Mesenchymal Cancer Cells on Tumor Plasticity and Metastasis

**DOI:** 10.3390/cancers16244158

**Published:** 2024-12-13

**Authors:** Nick A. Kuburich, Julia M. Kiselka, Petra den Hollander, Andrew A. Karam, Sendurai A. Mani

**Affiliations:** 1 Legorreta Cancer Center, The Warren Alpert Medical School, Brown University, Providence, RI 02912, USA; nick_kuburich@brown.edu (N.A.K.); juliakiselka@gmail.com (J.M.K.); petra_den_hollander@brown.edu (P.d.H.); andrew_karam@brown.edu (A.A.K.); 2 Department of Pathology and Lab Medicine, The Warren Alpert Medical School, Brown University, Providence, RI 02912, USA

**Keywords:** hybrid epithelial/mesenchymal, vimentin, keratin, cytokeratin, intermediate filaments, epithelial–mesenchymal transition, lung cancer, predictive biomarkers, targeted therapy

## Abstract

The epithelial–mesenchymal transition (EMT) program is critical to metastatic cancer progression. EMT results in the expression of mesenchymal proteins and enhances migratory and invasive capabilities. In a small percentage of cells, EMT results in the expression of stemness-associated genes that provide a metastatic advantage. Although EMT had been viewed as a binary event, it has recently become clear that the program leads to a spectrum of phenotypes, including hybrid epithelial/mesenchymal (E/M) cells that have significantly greater metastatic capability than cells on the epithelial or mesenchymal ends of the spectrum. As hybrid E/M cells are rarely observed in physiological, non-diseased states in the adult human body, these cells are potential biomarkers and drug targets. Hybrid E/M cells are distinguished by the co-expression of epithelial and mesenchymal proteins, such as the intermediate filament proteins cytokeratin (CK; epithelial) and vimentin (VIM; mesenchymal). Although these intermediate filaments have been extensively used for pathological characterization and detection of aggressive carcinomas, little is known regarding the interactions between CK and VIM when co-expressed in hybrid E/M cells. This review describes the characteristics of hybrid E/M cells with a focus on the unique co-expression of VIM and CK. We will discuss the structures and functions of these two intermediate filament proteins and how they may interact when co-expressed in hybrid E/M cells. Additionally, we review what is known about cell-surface expression of these intermediate filament proteins and discuss their potential as predictive biomarkers and therapeutic targets.

## Simple Summary

Hybrid epithelial/mesenchymal (E/M) cells are the most metastatic of carcinoma cell types. Hybrid E/M cells have both epithelial and mesenchymal properties reminiscent of chimeras, mythical creatures composed of different animals. The co-expression of epithelial and mesenchymal proteins is a defining feature of hybrid E/M cancer cells. In this review, we discuss the use of cytokeratin (epithelial) and vimentin (mesenchymal) intermediate filaments as markers in hybrid E/M cells. We describe the structures and functions of these filaments that are uniquely expressed in hybrid E/M cells and speculate on potential hybrid structures formed between vimentin and cytokeratin. We also describe cell-surface cytokeratin and vimentin and their functions and discuss their potential as predictive biomarkers and as targets for anti-cancer therapy.

## 1. Introduction

Carcinomas develop from epithelial tissues, known for their extensive cell-cell junctions that form sheets of cells and their stationary nature. The majority of carcinoma patients die due to the metastatic spread of the disease to distant organs [1], a process that requires cancer cells to be motile and invasive, which stands directly opposite to the stationary nature of epithelial cells. Carcinoma cells change from stationary (epithelial-like) to migratory (mesenchymal-like) due to activation of the epithelial–mesenchymal transition (EMT) program, which is typically reserved for embryonic development and tissue repair. Through the activation of EMT, carcinoma cells gain mesenchymal properties that are conducive to malignancy, such as invasion, motility, resistance to apoptosis, and stemness [2,3].

Although EMT was initially thought to be a binary transition of an epithelial cell to a mesenchymal cell, in physiological (embryonic development and wound healing) and pathological (chronic inflammatory tissue repair and carcinoma) contexts, activation of EMT results in cells with a spectrum of epithelial and mesenchymal properties, termed hybrid epithelial/mesenchymal (hybrid E/M) cells [4,5,6,7]. Critically, hybrid E/M cells are more tumorigenic and metastatically competent than cells at the epithelial and mesenchymal ends of the EMT spectrum [8,9,10,11]. Mutations or cell signaling events that induce primary tumors to adopt a hybrid E/M state accelerate tumor initiation and metastatic progression [9], and through single-cell lineage tracing, it was identified that metastatic competence peaks with hybrid E/M states [8,11]. These recent studies have positioned hybrid E/M cells as a critical cell type in metastatic carcinoma progression.

Due to the importance of EMT and hybrid E/M cells in metastasis, selectively targeting these cells is an attractive therapeutic strategy. The activation of the EMT program—and thus, the production of hybrid E/M cells—is temporally restricted to embryonic development and events such as wound healing or cancer [5]. Based on this information, we argue that hybrid E/M cells are rare in the adult human body, with the hybrid E/M cells being limited to normal cells in the body responding to inflammation, such as keratinocytes migrating to heal a wound or cancer cells activating EMT to gain metastatic properties. Thus, a hypothesized targeted therapy for hybrid E/M cells given to a carcinoma patient has the potential to selectively and specifically inhibit the most aggressive cancer cells driving metastatic dissemination. 

Developing biomarkers and targeted therapies fundamentally rely on the same concept: utilizing a specific marker or combination of markers that are uniquely and universally expressed in the cells of interest but not in other cell types. Thus, the development of such a biomarker or therapy relies on our understanding of what makes the hybrid E/M cells unique. In other words, we need to use markers that are present in the hybrid E/M cells that exist along the EMT spectrum, but not in the epithelial or mesenchymal cells that exist at the E or M termini. To this end, we assert that the co-expression of the epithelial cytokeratin (CK) and the mesenchymal vimentin (VIM) intermediate filament proteins are unique properties of hybrid E/M cells. Histopathologists have historically used epithelial-specific CK and mesenchymal-specific VIM for epithelial and mesenchymal characterization of cells in tissue sections [12,13,14]. Using this concept, cells can be described as epithelial if they express only CKs, as hybrid E/M if they express both CKs and VIM, or as mesenchymal if they express only VIM [9,11,15,16]. 

In this review, we first discuss the importance of EMT and hybrid E/M cells in carcinoma progression and expand on the concept that hybrid E/M cells are rare in adults. We then discuss the known structures and functions of the CK and VIM intermediate filaments and how the co-expression of CK and VIM leads to interactions, cytoskeletal dynamics, structures, and functions unique to hybrid E/M cells. Finally, we discuss the extracellular forms of these proteins, their function on the surface of cancer cells, and how these forms could be used to identify and target hybrid E/M cancer cells.

## 2. Physiological and Pathological Occurrences of Hybrid E/M Cells

Carcinoma progression begins with the tumorigenic transformation of a cell, followed by the expansion of this clone due to the unregulated growth, localized invasion into the surrounding tissue, metastatic dissemination, and the formation of metastatic tumors [17,18,19]. Metastasis leads to mortality in most carcinoma patients, as the metastasized tumors siphon critical nutrients from the organ that is the site of metastasis [1]. To escape from the primary tumor site, carcinoma cells need to release their stationary properties in favor of invasive properties. The abuse of the EMT program, mainly restricted to embryonic development and tissue repair, by these carcinoma cells allows them to escape from the primary tumor to become malignant and metastasize [20]. 

**Figure 1:**
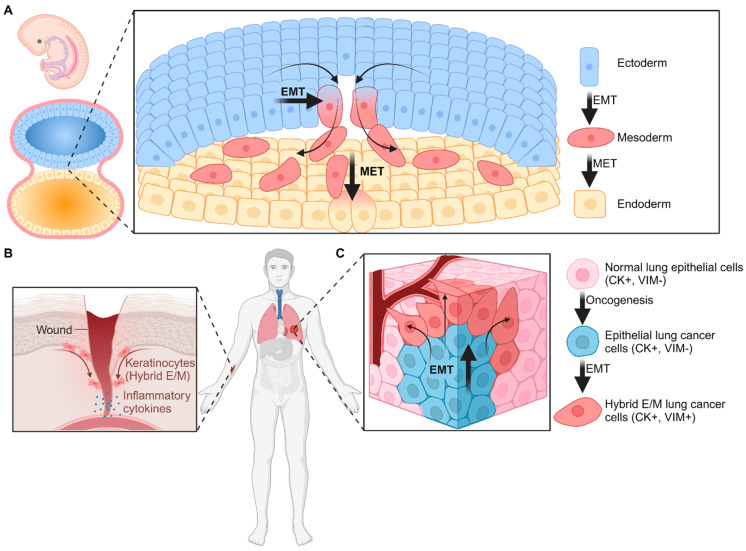
Hybrid E/M cells are rare in adults. (**A**) EMT occurs during embryogenesis, with the most notable examples being during gastrulation and the formation of the ectoderm, mesoderm, and endoderm from the epiblast cells. (**B**) EMT is also upregulated due to pathological events that lead to inflammation. During skin injury, for example, inflammatory cytokine production leads to EMT in keratinocytes. These mobile keratinocytes can migrate to the wound site to begin wound healing. (**C**) EMT occurs during cancer progression due to altered cell signaling or gene expression and leads to increased invasiveness and stemness. Abbreviations: EMT, epithelial–mesenchymal transition; MET, mesenchymal–epithelial transition; CK, cytokeratin; VIM, vimentin.

EMT is critical during embryonic development, as motility and invasiveness are necessary during the highly regulated steps of embryogenesis. For example, during gastrulation, EMT is required for the differentiation of the endoderm, mesoderm, and ectoderm germ layers (Figure 1A) [5,21,22]. During this stage, the EMT transcription factors Snail and Twist are critical regulators of EMT and are required for the establishment of the primary mesenchyme and gastrulation [23,24,25]. Meanwhile, EMT in adults is associated with inflammation and pathological events. The most common examples are tissue healing following a wound and fibrosis, with both contexts involving an inflammatory response [5,26]. In the context of a physical wound in the skin, basal keratinocytes, which express CK5 and CK14, near the basement membrane in the skin transiently upregulate EMT to facilitate wound closure and tissue repair (Figure 1B) [5,26,27]. The transcription factor Slug is required for EMT initiation and plasticity in keratinocytes following a wound [28]. Hybrid E/M cells are also observed during renal fibrosis through a Snail-driven partial EMT [29]. These contexts are the most prominent non-cancer-related instances of EMT upregulation and production of hybrid E/M cells and demonstrate the heterogeneity in EMT signal transduction pathways.

EMT is also induced in carcinoma cells, generating hybrid E/M cells that have a high propensity for tumor development and metastasis [9,10,11] (Figure 1C). As carcinomas originate from epithelial tissues, these cells are restrained to the primary tumor by their robust cell–cell adhesion and lack of migratory capacity. This epithelial nature is altered by EMT induction, which enables invasion into the surrounding tissue, the generation of cancer stem cells (CSCs), and the promotion of the metastatic spread to distant organs [2,3,4]. Once in a metastatic niche, epithelial properties are increased to enable cell–cell adhesion. Thus, epithelial–mesenchymal plasticity, which is the ability to modulate epithelial and mesenchymal properties to better adapt to the environment, is critical for cancer progression.

The output of the EMT program can differ based on the cellular context in which it occurs, leading to heterogeneity of the signal transduction pathways. For example, there are five reported distinct gene regulatory network sub-circuits during development, each regulated by different transcription factors that enable different functions [24]. This EMT variability also occurs in cancer, where the cellular history or hysteresis [30] can dictate the EMT transcription factors involved. For example, Snail-induced EMT is important in breast cancer metastasis [31], but Snail is irrelevant in pancreatic cancer metastasis, which instead relies on ZEB1 to drive EMT [20,32]. However, all of these instances utilize the EMT program to provide mesenchymal properties and regulate cellular differentiation states.

While the EMT program certainly has contextual differences, it is critical to note that a hybrid E/M state has been observed in all three of these EMT-involved contexts (development [24,33], wound healing/fibrosis [26,28,29], and carcinoma [8,9,10,11]), suggesting that these hybrid E/M cells and plasticity are critical. The EMT program is highly regulated in the human body, and each of the above three EMT-involved contexts is a discrete event. Development is temporally restricted to embryogenesis, wound healing/fibrosis is limited to tissue-damaging and inflammatory events, and carcinoma is, of course, restricted to cancer development. Thus, these EMT-involved contexts and the subsequently derived hybrid E/M cells appear to not be widespread in physiologically healthy adults. Because of this concept, hybrid E/M cells may be only present in the human body when it is related to these three contexts. While this idea must be further investigated and examined, if it is supported, it could suggest that hybrid E/M cells are an excellent marker of carcinoma cells. Furthermore, if a putative hybrid E/M targeted therapy was given to a carcinoma patient, it should selectively inhibit the hybrid E/M cancer cells, thus eradicating the most metastatic population [5,9,11]. While these ideas are exciting, they require an improved understanding of hybrid E/M cell biology to determine what targets are good for hybrid E/M cells and the expression profile of these markers in the body to characterize putative off-target effects. To this end, we will discuss how the co-expression of the two most used epithelial and mesenchymal markers, CK and VIM, could be used to identify and target hybrid E/M cells.

Before we continue, it is critical to discuss a potential issue when using CK and VIM as markers for hybrid E/M cells in vitro. Culturing cells in vitro leads to the upregulation of VIM and the acquisition of mesenchymal properties [34,35,36,37]. This is likely due to the drastic differences between in vitro cell culturing and physiological cell growth inside the body. The non-physiological conditions of culture (e.g., serum, growth factors, two-dimensional growth) could lead to the upregulation of VIM [34]. Therefore, the use of VIM and CK co-expression to identify hybrid E/M carcinoma cells should mainly be applied to in vivo models. Any in vitro work involving the use of VIM and CK co-expression should keep the potential gain of VIM during culturing in mind and be verified in vivo.

## 3. Co-Expression of CKs and VIM in Hybrid E/M Cancer Cells

**Figure 2:**
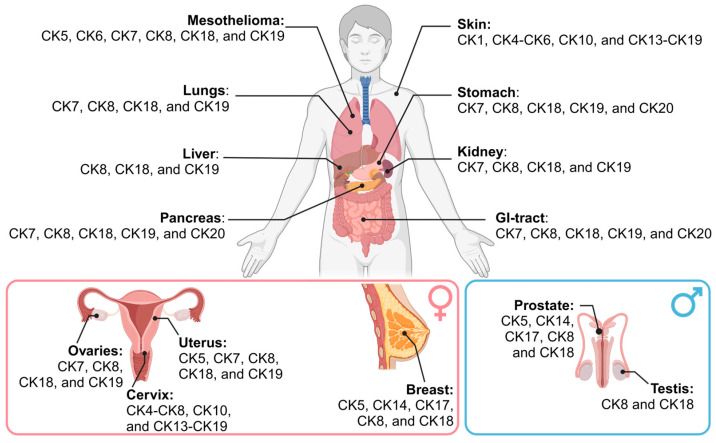
CK expression in human epithelial cells. CK profiles tend to be conserved between a given organ and a tumor originating from that organ, as determined by literature review [38,39,40,41,42,43,44].

The intermediate filaments CK and VIM are well-established markers of the epithelial and mesenchymal states, respectively. We have previously reviewed the similarities between CK and VIM, especially in the context of cancer cells [16]. Briefly, both proteins form intermediate filaments that, with actin microfilaments and tubulin microtubules, support the cytoskeleton. Whereas actin and tubulin are ubiquitous, the intermediate filaments differ depending on the cell type. Epithelial cells express CKs, whereas mesenchymal cells express VIM. CKs and VIM are highly related [16], but the CKs are far more complex due to the number of different CK proteins. The CK gene family consists of 54 different *KRT* genes, divided into Type I and Type II classes [38]. In comparison, VIM is produced from a single *VIM* gene, and this protein is categorized as a Type III intermediate filament. Consequently, different epithelial cell types can express different CKs for their cytoskeleton, while mesenchymal cells only have one option for VIM. Of the 54 *KRT* genes, about 21 of these *KRT* genes are epithelial cytokeratins (expressed in epithelial cells), and the remaining are either hair follicle-specific (expressed only in the root sheath) or hair and nail-specific [38,39]. Due to our focus on carcinomas and epithelial cells, we will focus on these 21 *KRT* genes that are expressed by epithelial cells and are associated with carcinomas. We have categorized the CK expression for different organs that develop cancer (Figure 2).

**Figure 3:**
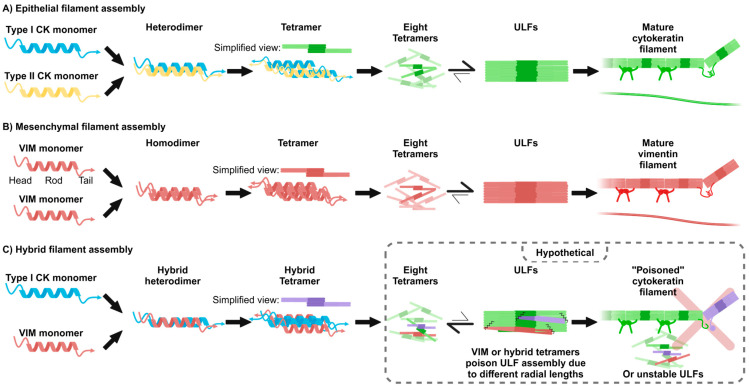
Assembly dynamics of CK and VIM filaments and hybrid filaments. (**A**) During CK intermediate filament assembly, a Type I monomer (blue) and a Type II monomer (yellow), in this example, CK14 and CK5, respectively, interact to form heterodimers, which interact to form heterotetramers (simplified as green), which interact to form ULFs. The ULFs then interact to form mature filaments in a process regulated by post-translational modifications. The equilibrium arrows between the eight tetramers and ULF denote that while this step is reversible, the reaction primarily proceeds toward ULF formation (larger arrow). (**B**) Assembly of VIM monomers (red) into mature filaments follows a similar mechanism to CK filament assembly. The equilibrium arrows between the eight tetramers and ULF denote that while this step is reversible, the reaction primarily proceeds toward ULF formation (larger arrow). (**C**) During hybrid filament assembly, a Type I CK (blue), in this example CK14, and VIM (red) interact through a conserved motif to form hybrid heterodimers and tetramers (simplified as purple). The hybrid tetramers are characterized as not having the stability to form ULFs or mature filaments, as visualized with squiggled lines and improper structure. The hypothesized result of this instability is that the vimentin/cytokeratin hybrid tetramers or vimentin/vimentin tetramers poison a cytokeratin ULF or halt mature filament assembly (denoted by the dotted gray rectangle in the bottom right). The equilibrium arrows between the eight tetramers and ULF denote that the reaction primarily proceeds to the disassembly and instability of ULFs (larger arrows) due to the reported incompatibility of hybrid tetramers with forming higher-ordered structures.

As intermediate filaments, CKs and VIM form dimers that then form tetramers; eight of these tetramers then assemble into unit-length filaments (ULFs) such that each ULF contains 32 monomers (Figure 3A,B). Filamentous structure formation and disassembly are regulated by phosphorylation, which has been extensively studied [45]. Intermediate filament proteins were first identified in 1968 [46]. However, the molecular architecture of mature intermediate filaments (composed of numerous ULFs) is still poorly understood, with the structures of the VIM tetramer and mature VIM intermediate filament being only recently resolved [47,48]. Although CKs and VIM assemble similarly into mature filamentous structures, there are critical differences between these intermediate filaments.

Epithelial cells usually express pairs of Type I CK and one Type II CK that hetero-oligomerize (Figure 3A). However, CKs can also interact and potentially hetero-oligomerize with other intermediate filament proteins, such as with CKs other than their pair or, interestingly, with VIM [49,50]. The profile of the CK pairs expressed in an epithelial cell can be used as a “fingerprint” during the histological identification of a tissue. For example, simple epithelial tissues (e.g., pleural lining in the lung) express CK8 and CK18 (Type II and Type I, respectively), whereas stratified epithelium tissues with luminal and basal cell types have cell-type dependent CK expression. For example, in breast tissue, the basal cells express CK5 and CK14, and the luminal cells express CK8 and CK18 (Figure 2) [51,52,53,54].

Carcinomas tend to maintain the CK expression profile of the tissue-of-origin; thus, characterizing the CKs expressed in a carcinoma can provide clues to the cancer type (Figure 2) [38,51]. For example, the lung’s luminal layer is made up of simple epithelium that predominantly expresses luminal CK8 and CK18 [38]; therefore, if a biopsy of a lung neoplasm reveals expression of CK5 and CK14, it could suggest a metastasis from a primary tumor such as basal-like triple-negative breast cancer, rather than a lung primary tumor. CK5 and CK6 staining has also been used to distinguish epithelial mesothelioma from lung adenocarcinoma or non-pulmonary adenocarcinomas [55], demonstrating the usefulness of characterizing CK expression in cancer.

Although carcinomas typically retain the CK expression profile of their tissue-of-origin, cancers do not necessarily keep that same CK profile during their progression. For example, CK switching can occur when the cells change their cytokeratin expression, such as a switch from luminal to basal CKs that is associated with oncogenic transformation and plasticity [56,57]. Some carcinoma cells are highly plastic, allowing them to adapt to different environmental factors and stressors; thus, if there is an advantageous phenotype that a cancer can adapt by altering its gene expression, then this phenotype could be selected [19]. For example, during the collective invasion of luminal breast cancer cells, the basal–epithelial program is upregulated in the leader cells with a gain of basal CK14 [58]. Depleting these cancer cells of CK14 is sufficient to block the collective invasion, demonstrating that CK14 is critical in this process [58]. Tumorigenesis can also lead to a change in CK expression compared to non-transformed cells. As an example, lung adenocarcinoma has been described as having increased levels of CK17 relative to normal lung tissues [59]. CK expression can also change during metastatic progression. In one report, a patient with lung cancer presented with a metastatic nodule on their neck that was CK7^−^/CK20^+^, leading to the diagnosis of small cell neuroendocrine carcinoma; however, postmortem analysis showed that the primary tumor was a small cell lung carcinoma that was CK7^+^/CK20^−^, suggesting that the CK expression profile switched during metastatic disease progression [60]. Another study characterizing lung primary tumors and metastatic nodule samples demonstrated that metastatic nodules frequently had higher levels of CK16 compared to the primary tumor [61]. Thus, it is critical to validate the cancer identity through biomarkers in addition to CKs, such as HER2, ER, and PR expression in breast cancer [62] and PSA in prostate cancer [63].

Like CK oligomerization, the filamentous assembly of VIM is regulated by phosphorylation of multiple sites located mainly in its amino-terminal head domain [64,65,66]. Phosphomimetic mutations of these residues (i.e., changing the serine to glutamic acid) or small-molecule administration (i.e., Withaferin-A or FiVe1) to stabilize these phosphorylation events results in an abnormal collapse of the VIM filaments leading to errors during mitosis [15,16,67,68,69]. Interestingly, inhibiting VIM assembly by stabilizing the phosphorylation of these key residues is detrimental to hybrid E/M cells, as it causes multinucleation and loss of stemness properties; however, it does not appear to adversely impact mesenchymal cells such as fibroblasts [15].

VIM can homo-oligomerize or hetero-oligomerize with nestin [70,71,72,73] or CKs [49,50] (Figure 3B,C). In migratory cells, VIM homo-oligomerization provides structural reinforcement and functions in signal transduction pathways, as previously reviewed [14,16]. VIM typically prefers to homo-oligomerize to form filaments, and its ability to hetero-oligomerize is both interesting and underexplored. Nestin obligately hetero-oligomerizes with VIM or the Type IV internexin [72]. Co-expression of nestin and VIM results in altered VIM filament dynamics during cell division, where VIM filaments collapse in the presence of nestin during mitosis [16,73]. Interestingly, VIM maintains its filamentous structure during mitosis in cells that lack nestin, where it binds with the actin–myosin cortex at the periphery of the dividing cell [16,74]. The functional implications of nestin-dependent VIM structural changes during interphase and how the cytoskeleton is altered in cells that co-express VIM and nestin are not well understood. Additionally, it is unclear how VIM deleterious disassembly in hybrid E/M cells due to FiVe1 treatment differs from the physiological VIM disassembly driven by nestin expression [15,68,73].

CK and VIM have been extensively studied to understand their highly dynamic and complex filamentous assembly and filament structures [47,48,50,75,76], but few studies have attempted to characterize how CK and VIM, occurring physiologically in hybrid E/M cells, assemble and function. There are several reasons for this gap in knowledge. First, the structural information for most intermediate filaments is surprisingly lacking, with only a few interactions contributing to intermediate filament assembly identified and the assembly process itself not fully elucidated. Second, the physiological co-expression of CK and VIM likely only occurs in hybrid E/M, which is restricted to development and pathological contexts. Most studies characterizing CK and VIM have used recombinant proteins or have used epithelial cells to study CKs and mesenchymal cells to study VIM [47,48,50,65,72]. Initial work on the assembly dynamics utilized cell-free assembly experiments with purified CK or VIM monomers, which can readily assemble into mature filaments [50]. These experiments were essential to further our understanding of intermediate filament structure and filamentous assembly. However, intermediate filament assembly in a cell can drastically differ from the assembly in a test tube due to a lack of factors found in the cell, such as other cytoskeleton members, linker proteins, kinases, and phosphatases, which could presumably impact the assembly dynamics.

Because of these reasons, several unknowns remain regarding the structure and functional changes that occur when CK and VIM are co-expressed in hybrid E/M cells. Regardless, the hybrid E/M cell type is pathologically critical for carcinoma progression, and the co-expression of CK and VIM in these cells is readily visible, warranting further examination and discussion [5,8,9,10,11,15,16]. To this end, we will discuss what is known regarding the structure and function when CK and VIM are co-expressed in hybrid E/M cells and provide our perspective to suggest how these traits could influence carcinoma progression.

## 4. VIM and CKs: Cooperators or Competitors?

The co-expression of CKs and VIM is a feature of aggressive carcinomas [16,77]. Melanoma cell lines that express both proteins are more metastatic and invasive than melanoma lines that express only VIM or lines depleted of CK8 and CK18 [78]. Breast cancer cells that express both VIM and CKs are more metastatic and invasive than cells without VIM [79]. Hybrid E/M cells co-expressing CK14 and VIM are more tumorigenic and metastatic than cells that express only CKs or only VIM [9,11]. Collectively, these studies support the hypothesis that VIM and CK co-expression is beneficial for cancer progression [16]. However, these studies discuss the outcomes of co-expression rather than characterize unique structures formed when these two intermediate filaments are co-expressed.

The first study to characterize VIM and CK co-assembly employed crosslinking experiments with purified proteins. In an equimolar solution of VIM and CK14, the assembly of VIM/CK heterodimers and heterotetramers that contained equal amounts of VIM and CK14 were detected alongside VIM/VIM and CK/CK dimers [50]. This finding suggests that a hybrid heterotetramer between VIM and CK can be formed. Furthermore, Steinert et al. verified that the hybrid heterotetramer was made up of a 1:1 ratio of VIM and CK14 through amino acid analysis of the bands [50]. They speculated that the formation of higher-order hybrid filament structures beyond the heterotetramer was unlikely due to differences between the proteins. VIM and CK14 have different axial lengths (43.9 nm for VIM and 46.2 nm for CK14), and the extent of stagger in antiparallel molecular alignments in tetrameric structures suggests that heterotetramers are incapable of assembling into higher-order structures [50]. Recent work using super-resolution imaging revealed that the axial length of VIM is longer than what was initially hypothesized from theoretical modeling of crosslinking experiments at ~49 nm [80]. Although this work did not calculate the axial length of CK, the authors suggested that the axial length of CK is likely longer than that of VIM due to the longer CK rod domain [80], supporting the hypothesis that heterotetramers cannot form higher-order structures due to structural incompatibility.

Steinert et al. also performed in vitro filamentous assembly experiments with different intermediate filament components to test this incompatibility hypothesis [50]. They characterized filamentous assembly over time using a light-scattering assay and observed that VIM monomers assembled rapidly into dimers, tetramers, and larger oligomers [50]. They then assessed if the addition of crosslinked dimers of VIM/VIM, CK5/CK14, or VIM/CK14 altered the reaction kinetics. The reaction occurred more rapidly in the presence of crosslinked VIM/VIM dimers, likely because these components bypass the need for dimer formation, thus speeding up the reaction [50]. Interestingly, in the presence of CK5/CK14 or VIM/CK14 crosslinked dimers, assembly into higher-order structures was inhibited [50]. Even when VIM monomers were incubated for 20 min to allow initiation of oligomer formation prior to the addition of crosslinked CK5/CK14 or VIM/CK14 dimers, the assembly into higher-order structures was inhibited [50]. The authors conclude that VIM filamentous assembly in vitro can be poisoned by the presence of CK-containing dimers [50]. Steinert et al. observed similar poisoning effects by VIM on CK filament assembly in the reciprocal assay [50]. CK5/CK14 crosslinked dimers accelerated the formation of oligomers of CK5 and CK14, and VIM/VIM or VIM/CK14 crosslinked dimers both inhibited the assembly of CK5/CK14 filaments [50].

In contrast to cell-free data that indicate that VIM and CKs do not form higher-order oligomers, varying levels of co-localization of VIM with CKs have been reported and verified through co-immunoprecipitation and confocal microscopy [49,79,81]. VIM and Type I CK14 interact in keratinocytes through a conserved sequence that is found in both VIM and Type II CKs [49,82,83] (Figure 3C). This conserved sequence, YRKLLEGEE, is located in the C-terminal helical 2B domain of VIM, spanning amino acids 400 to 408 [49,82,83]. In Type II CKs, this conserved sequence is required for interaction and polymerization with Type I CK, specifically for filament stabilization, but not filament elongation [83]. Mutations of this sequence in Type II CKs lead to diseases such as epidermolysis bullosa simplex, which causes severe blistering of the skin due to poor cytoskeleton assembly and cellular resilience [83,84].

Velez-DelValle et al. constructed a VIM mutation in this conserved sequence shared with the Type II CKs (i.e., replacement of the glutamic acid at position 405 of VIM with glycine, the mutation observed in epidermolysis bullosa simplex) and observed that it prevents the interaction between VIM and CK14 in keratinocytes [49]. This mutation prevents VIM co-immunoprecipitation with CK14 and results in decreased keratinocyte migration [49]. Expression of this VIM mutant also causes abnormal distribution of CK14 and VIM filaments [49]. Due to the known function of this mutation in CKs, this sequence is likely important for the assembly of heterotetramers, potentially due to the impact this mutation has on intermediate filament stabilization but not elongation [83]. Although the mechanism by which the CK/VIM interaction provides enhanced migratory capabilities was not characterized, the authors hypothesize that the ability of VIM to form the hybrid VIM/Type I CK dimers or tetramers would disrupt the rigidity of Type I/Type II CK filaments at focal points, increasing the migratory ability of the epithelial cells [49]. Velez-DelValle et al. proposed this hypothesis based on the poisoning effects between VIM and CK described by Steinert et al. [49,50].

The reported incompatibility of the VIM/CK heterotetramers with higher-order structures suggests that the expression of VIM during EMT could poison the established CK intermediate filament network, resulting in the transition from an epithelial cytoskeleton to a mesenchymal one. VIM might interact with the ends of the CK filaments at focal points in the cells, siphoning off Type I CKs from the Type II CKs to form chimeric heterotetramers that are unable to contribute to filament formation, functionally inhibiting filamentous elongation (Figure 3C). Supporting this hypothesis, studies assessing the co-localization and interaction between VIM and CK intermediate filaments identified focal points with more robust interactions with VIM [49,81]. The assembly and disassembly of intermediate filaments is a highly dynamic process, with annealing and fragmentation occurring simultaneously [76]; thus, newly expressed VIM could be incorporated into intermediate filaments composed of Type I and Type II CKs, causing instability. To our knowledge, this hypothesis has not been tested in hybrid E/M cells.

The logical extension of the poisoning hypothesis [49,50] is that VIM functions to overthrow the epithelial CK network during EMT. Supporting this, VIM has EMT-enforcing properties rather than just being a downstream structural protein produced by EMT. Exogenous expression or microinjection of VIM into epithelial cells is sufficient to induce a mesenchymal morphology with altered shape, adhesion properties, and desmosome localization [85,86]. Furthermore, expressing wild-type VIM in epithelial cancer cells leads to the upregulation of EMT transcription factors, Twist and Slug, whereas expression of a mutated VIM that is unable to form higher-order filamentous structures, does not increase expression of these EMT transcription factors, nor does it cause internalization of desmosomes [85,86]. The function of VIM in signal transduction may explain the upregulation of transcription factor expression, which suggests that VIM’s role in signal transduction is reliant on mature filament formation [13,86].

In a vacuum, the conclusion that VIM/CK heterotetramers are incompatible with higher-ordered structures and poison the assembly of VIM or CK filaments suggests that these two filaments are directly opposed to each other, constantly inhibiting the other’s assembly. However, in a hybrid E/M cell, it is clear that this is not the case, as both VIM and CK filamentous networks co-exist and co-localize with each other [10,49,79,81,85,86,87]. While the cell-free context does not invalidate the conclusions of the experiment [50], it is important to note that the co-localization of VIM and CK chimeric filaments observed in a cell could be formed due to factors that could improve filament stability, such as other cytoskeleton members, linker proteins, kinases, and phosphatases, which could alter the assembly dynamics. Thus, the poisoning action might contribute to filament dynamics in hybrid E/M cells, but widespread inhibition of the CK filaments due to EMT-induced VIM expression is not supported. VIM and CK might not be direct competitors; instead, their interactions at the heterotetramer level could dynamically influence the filamentous assembly in hybrid E/M cells and assist with the cytoskeletal changes during EMT.

Other studies characterizing the gain of VIM during EMT support the hypothesis that VIM and CK are not direct competitors. During the early stages of EMT in cultured neonatal rat hepatocytes, the newly synthesized VIM does not randomly form filamentous structures but rather follows the preexisting CK network [81]. Interestingly, these authors identified that while co-localization was observed extensively throughout the cell by immunofluorescence, higher-resolution immunoelectron microscopy revealed that CK and VIM filaments are closely interrelated but independent [81]. Specifically, co-localization was detected at discrete knots along the filamentous network. These discrete interactions likely explain the reports that VIM and CK co-localize at discrete sections in the cell and directly interact in co-immunoprecipitation assays [49], but also accounting for the reported incompatibility of higher-order hybrid intermediate filament structures [50].

The EMT-enforcing nature of VIM does not mean that the net outcome of VIM expression is the depletion of CK filaments [81,85]. The gain of VIM does not directly result in a decrease in endogenous CK expression; instead, VIM expression increases the number and thickness of overlaps or junctions of CK bundles (tonofibrils) [86]. The authors hypothesized that the thicker and tighter CK bundles induced by VIM expression function to increase the stress-enduring capacity of these cells during migration, where the CK filaments work synergistically with VIM to increase cell resilience [86,88]. Supporting this hypothesis, in lung cancer cells, VIM supports the pre-existing CK network following EMT [61]. In these cancer cells, VIM directly interacts with the Type I CK16, preventing FBXO21-mediated ubiquitination of CK16 and its proteasomal degradation, thus increasing CK16’s stability [61]. In another experiment that assessed the relationship between VIM and CK filamentous networks, antibodies that targeted either VIM or CK were microinjected into hybrid E/M cells [87]. Interestingly, the injection of either the VIM or CK antibodies independently led to the collapse of both filamentous networks [87]. However, injecting an anti-VIM antibody into an epithelial cell did not lead to CK network collapse, and injecting an anti-CK antibody into a mesenchymal cell did not result in VIM network collapse [87]. Together, these experiments support the idea that VIM and CK networks are co-dependent in hybrid E/M cells.

It remains to be determined if the structures and functions of the VIM and Type I CK interactions differ based on the CK protein expressed. Much like how CK monomers prefer to heterodimerize with specific CK counterparts, VIM may prefer to interact with certain Type I CKs over others, and these hybrid interactions may have different functions. Supporting this idea, cells expressing CK14 lead invasion during collective migration, and cells lacking CK14 but expressing the luminal CK8 and CK18 are the followers, demonstrating the different functions of the CK proteins [58]. Thus, there could be differences in assembly and function between VIM/CK14 and VIM/CK18 hybrid structures. Understanding the differences in assembly, structure, and function of hybrid filaments will be critical when leveraging co-expression as a biomarker or therapeutic target for hybrid E/M cancer cells.

To summarize, based on the currently available data, we hypothesize that VIM and CK filamentous networks co-exist in hybrid E/M cells, and these networks co-localize in the cytoskeleton but do not form higher-order hybrid CK/VIM structures. The VIM and CK monomers can form heterodimers and heterotetramers but are unlikely to form higher-order chimeric filaments. We speculate that the heterodimers and heterotetramers instead dynamically regulate filament assembly in hybrid E/M cells due to the poisoning effect [49,50] to support the EMT-induced cytoskeletal changes. Further, we speculate that VIM and CK interact in hybrid E/M cells to different degrees based on the cellular context and that this hybrid interaction enhances the resilience, elasticity, and strength of the cytoskeleton to accommodate stressors that are encountered when migrating.

## 5. Thinking Outside the Cell: Cell-Surface and Secreted VIM and CKs

**Figure 4:**
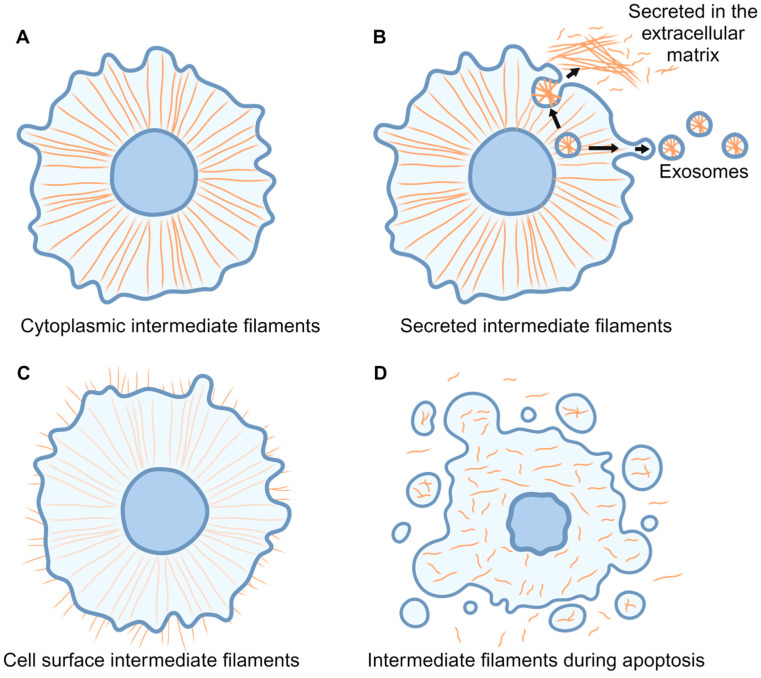
Localization of VIM and CKs inside and outside the cell. (**A**) Cytoplasmic intermediate filament proteins (IFs, orange) form a membrane-spanning filamentous network. (**B**) Cells can export intermediate filament proteins either by direct secretion into the extracellular matrix or through the release of exosomes containing these proteins. (**C**) Cell-surface VIM and CKs are detected on a variety of cell types but are most commonly associated with disease states such as infection or cancer. (**D**) Apoptosis can also lead to extracellular intermediate filaments. Filaments are disassembled during the early stages of apoptosis, and cell-membrane rupture or apoptotic blebs could result in extracellular intermediate filaments.

The focus of the prior sections has been on cytosolic intermediate filaments that span the cytoplasm from the nucleus to the cell membrane (Figure 4A). However, intermediate filaments are also detected on the cell surface and in the extracellular matrix, where they have functions distinct from those of their intracellular counterparts. The cell-surface localization of these filaments makes them accessible markers to potentially identify circulating carcinoma cells and provide a target for a hybrid E/M cell-selective therapy. The terms extracellular VIM and extracellular CKs describe the non-cytoplasmic localization of these intermediate filaments, either secreted outside the cell (Figure 4B) or localized to the extracellular cell surface (Figure 4C). Additionally, apoptosis could contribute to the production of extracellular intermediate filaments, as CK18 and CK19 fragments have been reported to be produced from apoptotic cells [89] (Figure 4D). The structures, functions, and translocation mechanisms of these extracellular intermediate filaments may differ depending on the cell type [90,91].

Critically, carcinomas are characterized to have higher levels of extracellular intermediate filaments than healthy tissues. A study seeking to identify putative biomarkers of small (≤2 cm) hepatocellular carcinomas showed that elevated serum VIM levels distinguished patients with and without these tumors [92]. A separate proteomic study to identify putative colon cancer biomarkers concluded that levels of extracellular VIM were significantly higher in the serum of these patients [93]. The utilization of cell-surface CK to identify carcinoma cells was first reported in 1991 when CK8, CK18, and CK19 were detected as aggregates on MCF-7 breast carcinoma cells but not normal mammary cell lines in primary cultures [94]. Subsequent work verified that CK8 was also found on the cell surface of head and neck carcinoma cells but not on healthy squamous epithelial cells [95], and that CK8 was detected on the cell surface of prostate cancer cells but not on normal prostate cells [96]. Extracellular CK1 is also upregulated in a variety of cancers, such as hepatocellular carcinoma, nasopharyngeal carcinoma, neural blastoma, and breast cancer cells [97]. Prostate, squamous cell carcinomas, and head and neck carcinoma cell lines have higher levels of cell-surface CK8 relative to non-tumor cell lines [95,96]. Furthermore, lung cancers secrete CYFRA 21-2, a CK19 fragment found in the serum of lung cancer patients (Figure 4B). CYFRA 21-2 has been leveraged as a diagnostic marker of non-small cell lung cancer and as a biomarker to predict objective response and survival in these patients [98,99,100,101,102].

**Figure 5:**
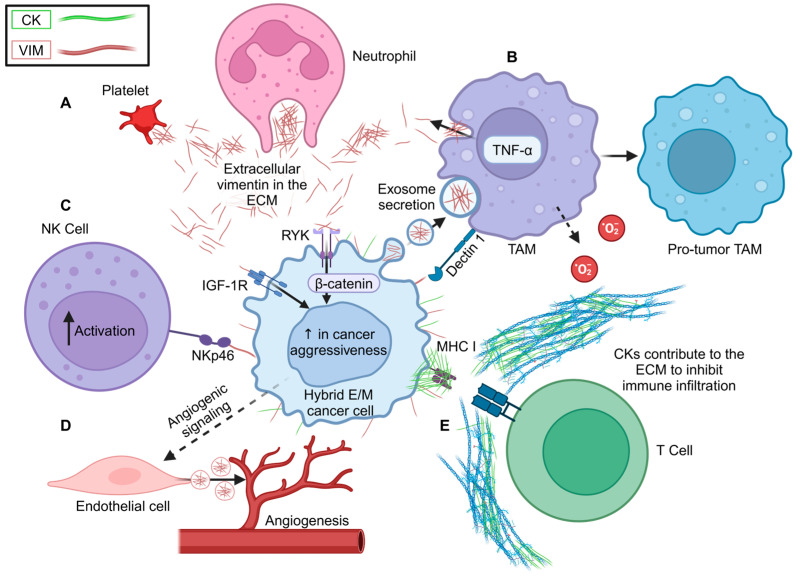
Multifunctional roles of extracellular CKs and VIM. (**A**) A variety of cells secrete intermediate filament proteins into the extracellular matrix (ECM) as a result of inflammation or cancer progression. (**B**) Cancer cells secrete VIM in extracellular vesicles, which can polarize macrophages to an anti-tumoral phenotype, and an increase in TNF-α exacerbates VIM secretion by macrophages. Direct binding to cell-surface VIM through dectin 1 promotes the release of the superoxide anion by macrophages. (**C**) NK cells interact with cell-surface VIM, leading to an increase in cytotoxic activity. However, this was described in bacterial infection, and the functional outcome of extracellular vimentin and NK cell interaction is not understood in carcinomas. (**D**) Angiogenic signals produced by carcinoma cells lead to an increase in extracellular VIM production by endothelial cells and an overall increase in angiogenesis. (**E**) Cell-surface CKs promote immune evasion by masking MHC I on cancer cells or by altering the ECM to prevent immune infiltration.

The described increase in extracellular intermediate filaments in the tumor microenvironment can also be produced by non-cancerous cells. Extracellular VIM is produced by macrophages, neutrophils, endothelial cells, platelets, fibroblasts, and other cells, where it is involved in cell signaling, carcinomas, inflammation, senescence, stress, and responses to viral or bacterial infections or wound healing (Figure 5A) [90,91,103,104,105,106,107,108,109,110,111,112]. Extracellular secretion of VIM has been reported to be mediated by the kinase PKC in macrophages [91,103]. IL-10-mediated inhibition of PKC activity prevents secretion of VIM, whereas TNF-α-mediated activation of PKC leads to an increase in secreted VIM from macrophages (Figure 5B) [103]. VIM phosphorylation by PKC regulates integrin trafficking to the cell surface in mouse fibroblasts; thus, VIM could translocate to the membrane through this mechanism [113]. Additionally, cancer cells produce exosomes that contain VIM, and these exosomes are taken up by tumor-associated macrophages (TAMs), inducing cytoskeletal rearrangement and polarization of the TAMs, making them tumor-supporting (Figure 5B) [114]. Cell-surface VIM has been identified as a putative target for natural killer (NK) cells to identify and inhibit infected cells [115]. This cell-surface VIM binds to the NKp46 receptor on the NK cells, leading to increased lysis of the infected cells [115] (Figure 5C). Thus, cell-surface VIM on cancer cells could modulate NK cell activity; however, this needs to be further characterized (Figure 5C).

Tumor-associated endothelial cells can be another source of extracellular VIM (Figure 5D). These endothelial cells secrete VIM in response to pro-angiogenic signaling from the cancer cells, and angiogenesis inhibitors prevent the secretion of VIM [116]. Tumor-associated endothelial cells actively secrete VIM through pro-angiogenic pathways through the type III unconventional protein secretion mechanism [116]. This extracellular VIM functions to promote a pro-angiogenic phenotype by binding to the VEGF receptor to mimic and upregulate VEGF signaling [116]. The authors identified that tumor-associated endothelial cells secrete VIM through the unconventional type III protein secretion mechanism [116]. This observation parallels the extracellular CK secretion that occurs in endothelial cells following oxidative stress, where cell-surface CK1 is expressed [117]. The production of extracellular VIM and CK from immune and endothelial cells, which are both involved in carcinoma progression, could contribute to the higher levels of extracellular intermediate filaments in the serum of cancer patients relative to healthy donors [92,93,96,97,98,99,100].

Secreted VIM is proposed to be a ligand for several different receptors. For example, the insulin-like growth factor receptor IGF-1R, which is overexpressed in cancer cells, binds to extracellular VIM [118,119]. IGF-1R is known to be involved in carcinoma progression: it promotes tumor initiation and metastatic seeding, especially in lung cancer [120]. To specifically test if the extracellular VIM/IGF-1R interaction occurs in carcinomas, breast cancer cells or normal breast cells in culture were treated with recombinant VIM with or without IGF-1R inhibitors [119]. Interestingly, there was a pronounced increase in proliferation, migration, and adhesion due to the upregulation of IGF-1R-mediated signaling in the breast cancer cell line relative to the normal breast cell line, and this increase was reversed upon blocking IGF-1R [119]. These results suggest that carcinoma cells could utilize extracellular VIM as a ligand to drive IGF-1R downstream signals; however, more work is needed to establish this, to determine if it occurs in other cancer types, and to characterize the functional outcomes of extracellular VIM-mediated signaling.

The surface-expressed pattern-recognition receptor dectin-1 also binds to extracellular VIM [121]. Dectin-1 is mainly expressed on macrophages and is involved in pathogen recognition, respiratory burst, phagocytosis, and cytokine and chemokine production. VIM binds to dectin-1 expressed on macrophages, leading to excessive inflammation due to the production of NADPH oxidase-derived superoxide anion (Figure 5B) [121]. Extracellular VIM is also involved in regulating the dendritic cells of the adaptive immune system [110]. Extracellular VIM decreases the lipopolysaccharide-mediated activation of dendritic cells and decreases the secretion of the pro-inflammatory cytokines IL-6 and IL-12 while increasing the secretion of the anti-inflammatory cytokine IL-10 from dendritic cells [110]. Given these findings, it is plausible that VIM secreted by cancer cells suppresses the anti-cancer immune response.

For cancer cells in culture, the addition of recombinant VIM led to an increase in invasiveness through the upregulation of Wnt signaling and β-catenin activity, which is involved in multiple critical cell signaling pathways, including EMT and proliferation [122,123,124,125]. In this study, colon cancer cell lines were cultured with exogenous VIM in the media and then characterized for changes in signaling pathways [122]. Interestingly, the extracellular VIM bound to and remained on the cancer cells, where it activated the Wnt signaling pathway with an increase in nuclear β-catenin and an upregulation of Wnt/β-catenin downstream targets [122]. Functionally, the extracellular VIM led to an increase in the invasion of these colon cancer cell lines. The authors proposed that extracellular VIM binds to the receptor tyrosine kinase RYK to upregulate the Wnt/β-catenin signaling pathway (Figure 5A) [122]. Thus, this study provides a signal transduction mechanism of how extracellular VIM can directly upregulate the aggressive properties of carcinomas.

Cell-surface CKs also appear to support carcinoma progression by upregulating immune evasion and chemoresistance. CK8, CK18, and CK19 expressed on the surfaces of UP-LN1 cells, a metastatic head and neck cancer cell line, inhibited the interaction between the MHC-I and T cell receptors by functionally masking the MHC-I molecules on the cancer cell surface and preventing their recognition by T cells (Figure 5E) [126]. MHC-I is a cell-surface molecule responsible for binding peptides derived from pathogens and presenting them to the appropriate T cells, and cancer cells frequently downregulate or mask MHC-I [127]. Thus, cell-surface CK8 could lead to immune evasion by carcinoma cells. Additionally, cell-surface CK8 expression is associated with increased multidrug resistance, and depletion of CK8 leads to increased chemotherapy sensitivity [128]. Cell-surface CK1 is also implicated in chemotherapy resistance [129]. Here, the nasopharyngeal carcinoma cell line CNE2 became resistant to cis-diamminedichloroplatinum chemotherapy when cells were cultured with increasing concentrations of the drug over time, and then the researchers sought to characterize changes in protein expression in the resistant clone relative to the parental cell line [129]. Cell-surface CK1 was expressed at higher levels on resistant cells than the original cells, and depletion of CK1 led to a decrease in chemotherapy resistance [129]. As the mechanism of this resistance has not been characterized, it is unclear if cell-surface CK expression is associated with resistance or actively promotes it.

The development of antibodies specific to extracellular intermediate filament proteins has enabled research into their application to detect CTC and the development of exciting therapeutic strategies [97,130,131]. Antibodies that bind to cell-surface VIM have been effectively used to detect CTCs in patients with gastric cancer [132,133], sarcoma [134,135], glioblastoma [131], neuroblastoma [136], hepatocellular carcinoma [137], breast cancer [138], prostate cancer [139], and lung cancer [140]. These studies across multiple cancer types demonstrate the application of using cell-surface VIM as a biomarker for CTCs. We postulate that combining an anti-cell-surface CK antibody with an anti-cell-surface VIM antibody would be an excellent strategy for detecting CTCs, especially those from hybrid E/M carcinomas. However, the multiple types of CKs that carcinomas can express can lead to issues with this approach. For example, an antibody specific to cell-surface CK1 may not be applicable to other cell-surface CKs depending on the antigen it recognizes. A pan-cell-surface CK antibody could solve this issue. For example, in characterizing CTCs, combining the anti-cell-surface VIM antibody with a pan-CK antibody led to higher detection of carcinoma CTCs than the combination of anti-cell-surface VIM and anti-CK7/8 antibody [141]. A pan-anti-cell-surface CK antibody will likely be necessary, as carcinomas with high levels of dedifferentiation are able to modulate their CK expression [142]. However, using a specific cell-surface CK antibody could provide increased specificity for identifying specific types of carcinomas.

Interestingly, the antibodies to cell-surface intermediate filament proteins have impressive anti-tumor effects on their own. Treating multiple glioblastoma cell lines in culture with anti-cell-surface VIM antibody 86C led to increased apoptosis in a dose-dependent manner [131]. The antibody was internalized within 15 min of treatment, and the levels of phosphorylated ribosomal protein RPS6 were dramatically reduced by treatment with the anti-cell-surface VIM antibody [131]. The RPS6 pathway is involved in multiple functions, including protein synthesis, proliferation, invasion, and apoptosis resistance; thus, the decrease of RPS6 phosphorylation could explain the increase of apoptosis in these cells [131]. Importantly, these researchers also showed that 86C has anti-tumor efficacy in vivo: mice with glioblastoma tumors treated with 86C had smaller tumors and increased survival compared to control mice [131]. A subsequent study characterized the potential of combining 86C with temozolomide (TMZ), the standard of care for glioblastoma, and showed that treating glioblastoma cells with 86C led to TMZ sensitivity in previously resistant cell lines [143]. Additionally, the combination led to enhanced tumor cell apoptosis and increased survival of mice with glioblastoma tumors compared to either agent alone [143]. Regarding the mechanism of action for anti-cell-surface VIM therapies, it appears that CSCs have more cell-surface VIM expression, as most cell-surface VIM-expressing cells also expressed the glioblastoma CSC markers, CD133 or CD44 [131]. The association between cell-surface VIM and CSCs was also demonstrated in hepatocellular carcinoma cells [137]. It is well known that CSCs are a major driver of cancer resistance [3]. Thus, one could hypothesize that the mechanism of action for cell-surface VIM therapy in this context is to bind to cell-surface VIM and induce apoptosis of CSCs, leading to increased TMZ sensitivity [143]. The effects of inhibiting extracellular VIM on carcinoma progression have also been evaluated by injecting mice with anti-VIM antibodies and by immunizing mice against VIM. Both approaches led to an increase in anti-tumor immune responses, decreased angiogenesis, and decreased tumor growth [116].

Antibodies targeting cell-surface CKs have also been explored as an anti-cancer strategy. In one study, mice were immunized with MCF7 breast cancer cell lysates, and the antibodies raised by the mouse were characterized [144]. The main antibody produced from this immunization recognizes a conserved epitope on CK1, CK2, CK8, CK10, and CK18 [144]. Treating these breast cancer cells with this antibody led to a decrease in invasiveness due to inhibited plasminogen activation; plasminogen is critical for invasion, as it degrades the extracellular matrix [144,145]. Researchers have also leveraged a 10-mer linear peptide termed 18-4 that specifically binds to cell-surface CK1 to develop a peptide drug conjugate [146]. The 18-4 peptide was conjugated to doxorubicin to target this chemotherapy to breast cancer cells [146]. Mice injected with MDA-MB-231 human breast cancer cell lines and then treated with this conjugate had reduced tumor growth over time relative to mice treated with vehicle or with unconjugated doxorubicin [146]. The peptide–drug conjugate also had more optimal biodistribution than free doxorubicin [146]. To our knowledge, peptides or antibodies that specifically bind to cell-surface VIM have not been used to deliver chemotherapy, but this approach would likely have high efficacy as well.

A unique cell-surface VIM-based therapy involves the cowpea mosaic virus (CPMV), which infects the leaves of the cowpea plant [147]. Cell-surface VIM is used for cell attachment mechanisms for multiple viruses, including CPMV [148]. Even though CPMV is a plant pathogen that does not infect human cells, the pathogen recognition receptors (PRRs) utilized by the immune system still identify it as a virus that needs to be eradicated [149]. Cell-surface VIM-positive cells (including cancer and immune cells) selectively bind and internalize CPMV, while cells without cell-surface VIM did not take up the virus [147]. Interestingly, the binding and internalization by immune cells and cancer cells both function to drive the immune response. Following the uptake, the toll-like receptors (TLR2, TLR4, and TLR7) class of PRRs are activated to upregulate immune signaling pathways to activate, recruit, and polarize immune cells to anti-tumor phenotypes [150,151]. CPMV treatment polarizes neutrophils to be more immunostimulatory, producing cytokines and chemoattractants to raise an inflammatory response [152]. Dendritic cells and macrophages that take up CPMV also produce increased pro-inflammatory cytokines [152,153]. Additionally, the macrophages infiltrate the tumor immune microenvironment and are polarized to be M1 macrophages (more anti-tumor function) [150]. Critically, CPMV has excellent homing to the tumor and biodistribution when administered to tumor-bearing mice with minimal leeching/distribution to other organs [154]. The mechanism of the anti-tumor effect by CPMV therapies is still being characterized. However, experiments support that the mechanism of action is dependent on the immune system, as the anti-cancer and anti-metastatic effects of CPMV were abrogated in immunocompromised mice and when neutrophils were depleted [152]. CPMV does not directly display tumor cell cytotoxicity but rather drives an anti-tumor response and facilitates immune cell infiltration to the tumor, effectively turning a cold tumor hot [152,155]. The CPMV-mediated anti-tumor effect has been demonstrated in melanoma, glioma, ovarian, colon, breast, and lung murine cancer models in vivo [152,156,157]. CPMV has been reported as safe in murine models, as it does not infect mammalian cells, does not lead to de novo viral RNA, and is not toxic to human blood or immune cells [149].

Another strategy utilizing cell-surface VIM as a facilitator of anti-cancer therapies involves IL-12. This cytokine has promising anti-tumor effects due to its activation of the innate and adaptive immune response [158]. However, IL-12 was found to have dose-limiting toxicities in clinical trials due to the presence of excessive systemic IL-12 [159]. To circumnavigate this, researchers constructed CAR-T cells that expressed a cell-membrane anchored IL-12 fused with a C-terminal peptide VNTANST that binds to cell-surface VIM. This peptide targets the CAR-T cells to tumors that express cell-surface VIM [160]. These CAR-T cells have robust anti-tumor effects in melanoma and osteosarcomas in mice resulting from enhanced T cell infiltration and IFNγ production [160,161]. Critically, this strategy prevents IL-12-associated toxicity with fewer histological changes in the livers of the mice, no T cell accumulation of non-cancerous organs, and lower serum concentrations of IL-12 compared to treatment with free IL-12 [160]. However, targeting VIM is not just limited to the cell surface, as targeting the intracellular, cytoplasmic VIM using withaferin-A or FiVe1 also results in robust anti-tumor responses [67,68,162,163,164]. We have previously reviewed the potential of targeting VIM phosphorylation using these small molecule strategies in more detail [16].

In summary, cell-surface CK and VIM are associated with inflammatory events such as viral and bacterial infections, wound healing, and carcinoma. Although cell-surface CK and cell-surface VIM have potential as biomarkers and in targeted therapies, more research is needed to understand the biology of these cell-surface intermediate filament proteins. Further work is needed to characterize combining cell-surface CK and cell-surface VIM-mediated strategies (e.g., antibodies, drug conjugates, or CPMV) for both the detection and inhibition of hybrid E/M cells that express these proteins. Additionally, the safety profile of targeting the extracellular CK and VIM, which is present on immune cells, must be verified. However, it should be noted that approaches such as using CPMV have shown little to no toxicity and a lack of viral infection in murine models [149].

## 6. Conclusions and Future Perspectives

Multiple publications support the idea that carcinoma cells exist along an E/M spectrum [6,7,8,9,10,11]. These studies led to a paradigm shift in the field from viewing the outcome of EMT as mesenchymal cells to the understanding that EMT results in cells with varying levels of epithelial and mesenchymal properties. We now understand that hybrid E/M cells disproportionately contribute to metastasis and carcinoma progression. There is a pressing need to identify what distinguishes hybrid E/M cells from the pure epithelial or mesenchymal carcinoma cells in the heterogeneous tumor population, requiring a systematic characterization of these cell states. Characterizing the traits specific to hybrid E/M cells will lead to biomarkers and will guide the development of strategies to target vulnerabilities unique to these cells. Co-expression of the epithelial CK and the mesenchymal VIM in vivo appears to be a trait specific to hybrid E/M cells. These intermediate filament proteins have been extensively studied since their identification as a significant component of the cytoskeleton in 1968 [46], and expression of these proteins is used to distinguish epithelial from mesenchymal cells. These proteins share a general mechanism of filamentous assembly and have similar structures. Both function to bolster the cytoskeleton, rely on phosphorylation of the head domain for assembly, have conserved interaction domains in their tails, and have similar cellular localization. CK and VIM differ in expression profiles, genetic diversity (there are 54 CKs in humans and one VIM), and assembly dynamics (i.e., CKs only hetero-oligomerize, whereas VIM can form homo- and hetero-oligomers).

Most of the research on CKs and VIM has understandably been focused on their structures and functions in epithelial or mesenchymal cells, respectively, leading to knowledge gaps when they are co-expressed in hybrid E/M cells. Both VIM and CK are incorporated into the cytoskeleton of hybrid E/M cells with considerable co-localization [10,49,79,81,85,86,87]. The CTCs that have the highest metastatic potential are those cells that co-express VIM and CK [9,11]. VIM can interact with Type I CKs to form heterotetramers; however, there are many unknowns regarding the structures and functions of these hybrid molecules. It is unclear if the interaction only occurs at the heterotetramer level due to incompatibility in forming higher-order filamentous structures, resulting in poisoning of the CK filaments, or if higher-order chimeric filaments are present in hybrid E/M cells. The reported co-localization could reflect higher-order hybrid filament structures or could be related to their shared localization in the cytoskeleton with discrete focal interactions rather than true hybrid VIM/CK filaments. Work on the structure and functions unique to hybrid E/M cells co-expressing CK and VIM is required.

Both VIM and CKs are detected on cell surfaces, in exosomes, and in the extracellular matrix during inflammation and cancer progression and have potential as biomarkers or targets of cancer therapy. Extracellular intermediate filament proteins have been used as biomarkers to identify CTCs from a variety of cancer types [138,139,140], including in lung cancer patients [61,89,98,99,100,102], and as an actionable target for anti-cancer therapies [131,143,144,147,152,156,157]. We expect that future research on the translocation mechanisms, structures, and functions of these extracellular intermediate filaments will improve our understanding of their functions and will lead to their application in carcinoma treatment.

It should be noted that VIM and CKs are not the only co-expressed epithelial and mesenchymal proteins in hybrid E/M cells. E-cadherin and N-cadherin are also epithelial and mesenchymal counterparts that are both expressed by these highly plastic cells. Other factors can also be leveraged to characterize hybrid E/M cells, including the mesenchymal EMT-inducing transcription factors (e.g., Snail, ZEB1, FOXC2, Twist) and the epithelial E-Cadherin, ESRP1, and EpCAM. The consensus statement for guidelines in the EMT field encourages the use of multiple markers to define EMT states rather than relying on a single marker [4], which we encourage as well. However, we propose that CK and VIM are two excellent markers that should be utilized when characterizing hybrid E/M cells. Work characterizing the co-expression of epithelial and mesenchymal intermediate filament proteins will be critical in developing biomarkers and selective methods for targeting hybrid E/M carcinoma cells.
